# The Impact of Allergic Rhinitis on the Quality of Life Among Adult Patients in Alahsa, Saudi Arabia: A Case-Control Study

**DOI:** 10.7759/cureus.87733

**Published:** 2025-07-11

**Authors:** Abdulhameed B Al Khalaf, Mohammed Khalid J Aljerayed, Mudhawi Alsuliman, Sajjad Naji Ahmed Alkhalifah, Sajidah F Alghazal, Hasan M Alhaddad

**Affiliations:** 1 Family Medicine, Alahsa Health Cluster, Alahsa, SAU; 2 General Practice, Alahsa Health Cluster, Alahsa, SAU; 3 Adult Allergy and Immunology, Almoosa Specialist Hospital, Alahsa, SAU

**Keywords:** allergic rhinitis, ghq-12, health-related quality of life, insomnia, saudi

## Abstract

Background and objective

Allergic rhinitis (AR) is highly prevalent in Saudi Arabia, yet its broader consequences on adult well-being remain underexplored. In this study, we aimed to compare health-related quality of life (HRQoL), psychological distress, and insomnia between adults with and without AR in Alahsa, Saudi Arabia.

Methods

A retrospective case-control study was conducted from December 2024 to May 2025. Fifty adults (≥18 years) with the International Classification of Diseases, 10th revision (ICD-10)-confirmed AR were randomly selected from Al Jabr Eye and ENT Hospital records; 150 controls without AR or chronic illness were recruited from the general community (1:3 ratio). Participants completed the General Health Questionnaire-12 (GHQ-12), Athens Insomnia Scale (AIS), and mini Rhinoconjunctivitis Quality of Life Questionnaire (miniRQLQ). Between-group differences were analysed with χ² or independent t-tests; Pearson correlations were employed to assess associations among scales.

Results

Cases and controls were comparable in age (mean age: 35.5 ± 11.2 vs. 34.6 ± 11.6 years, respectively) and sex distribution [23 males (46%)]. A significant difference was noted in educational level (p=0.023). AR cases scored significantly worse on all GHQ-12 items (p<0.001); 19 (38%) met the threshold for mild-to-moderate psychological distress vs. eight (5%) among controls. Clinically relevant insomnia (AIS ≥6) was present in 39 (78%) of cases and 34 (23%) of controls (p<0.001). Mean miniRQLQ domains most affected were tiredness/fatigue (2.98 ± 1.73) and sleep difficulty (2.80 ± 1.98). AIS scores correlated moderately with miniRQLQ scores in cases (r=0.40, p=0.006).

Conclusions

Based on our findings, AR imposes substantial burdens on mental health, sleep quality, and daily functioning among Saudi adults. Multidisciplinary management, including behavioural sleep counseling and psychological support, should accompany pharmacotherapy to reduce the societal impact of AR.

## Introduction

Allergic rhinitis (AR) is considered one of the most common diseases in the world. It is a chronic upper airway inflammatory disease involving multiple different cells and cytokines, mainly mediated by immunoglobulin E (IgE) produced by plasma cells after allergen exposure. Typically, AR manifests as frequent attacks of nasal symptoms, including sneezing, runny nose, nasal itching, and nasal congestion, which seriously affect the quality of life. The incidence of AR, as shown in epidemiological surveys, is about 20-40%, reaching as high as 50% in some countries [1]. AR is one of the world's most significant public health issues [2]. A study done in Korea by Kim et al. reported that the prevalence of AR was 15-25% across Asia, Europe, the Americas, and Africa [3]. Its prevalence is significantly on the rise in Saudi Arabia [4]. A study from Saudi Arabia by Alreshidi et al. involving 900 Saudi adults reported an AR prevalence of 34% [5]. In Al-Madinah Al-Monawara population of Saudi Arabia, the prevalence was 27.9% among 524 sampled participants [6]. Due to its recognizable prevalence, researchers have shown increasing interest in its effects on the quality of life of many patients and their families.

Several studies have emphasized that an individual's social environment, personal experiences, and relationships play a crucial role in shaping their perceived quality of life. According to the World Health Organization (WHO), quality of life refers to how individuals perceive their position within the cultural and value systems in which they live, in relation to their goals, expectations, standards, and concerns. Scholars commonly categorize quality of life into four major dimensions: (1) physical well-being, which includes aspects like health, nutrition, leisure activities, mobility, and the capacity to perform daily tasks; (2) emotional well-being, encompassing feelings of happiness, personal security, spirituality, self-esteem, and the absence of stress or fear; (3) social well-being, which relates to personal relationships, social support, community roles, work environment, and social status; and (4) material well-being, involving socioeconomic status, safety, employment, and access to rights and resources [7].

Patients view AR as a minor inconvenience rather than a disease that mildly to severely impairs the quality of life. Patients commonly experience sleep disturbances, poor interpersonal relationships with family and peers, mood swings, and easy fatiguability, which leads to decreased productivity at work and poor academic performance. Hence, a disease that has minimal treatment costs ends up putting a significant economic burden on society due to the non-medical losses that occur due to untreated disease [8]. Several diverse studies have been conducted to assess the quality of life in patients with AR. One of the most extensive studies involves a systematic review and meta-analysis in China by Liu et al. It assessed the associations between AR and patients' sleep duration and sleep issues. The study involved 240,706,026 patients (19,444,043 with AR). It demonstrated that AR was associated with daytime dysfunction, which includes daytime sleepiness, headaches, and difficulty waking up. It shed light on AR patients' need to use sleep medications on multiple occasions in their lives. In brief, the study supports the association between AR and sleep disturbances [9].

In 2019, a case-control study conducted by Loannis et al. in Greece explored the impact of AR on patients' quality of life. The study included 103 individuals diagnosed with AR confirmed by skin prick testing and compared them to 50 control participants without a history of allergy or related symptoms in the preceding year. To assess quality of life, the researchers used three validated tools: the General Health Questionnaire-28 (GHQ-28), the Athens Insomnia Scale (AIS), and the mini Rhinoconjunctivitis Quality of Life Questionnaire (miniRQLQ). The results showed that AR patients scored significantly higher on the GHQ-28, indicating greater psychological and social impairment compared to controls. AIS results further revealed that sleep quality was consistently poorer among AR patients, with elevated scores across all subdomains. Similarly, findings from the miniRQLQ indicated that individuals with AR experienced greater disturbances across multiple areas, including physical and social functioning, nasal and ocular symptoms, sleep quality, and general well-being, highlighting a significantly lower quality of life in the AR group [10].

In 2023, a study by Alwesaibie et al. conducted in Alahsa, Saudi Arabia, examined the relationship between AR and stress levels. The findings showed that 70.9% of participants experienced a moderate to high impact of AR symptoms on their daily activities. Disturbed sleep quality was reported by 67.3% of AR patients. Regarding psychological stress, 57% demonstrated moderate levels, 18.7% reported high levels, and only 10% were free from stress symptoms. Nasal obstruction (61.4%) and sneezing (59%) were the most common nasal complaints, while eye-related symptoms such as lacrimation (49.4%) and itching (44.2%) were also frequently reported. Notably, among individuals who experienced AR symptoms more than three times per week, 29.1% exhibited high stress levels [11]. A study by Alhazmi et al. in Saudi Arabia to check the prevalence and risk factors of chronic rhinosinusitis, involving 4,963 individuals in Saudi Arabia, revealed a 22.5% prevalence of chronic rhinosinusitis among Saudi individuals. Moreover, it also established that chronic rhinosinusitis negatively impacted the participants' quality of life [12,13].

In light of this, it is crucial to have a clear understanding of the impact of AR on the patient's quality of life and how the disease affects their physical, mental, and social development. It may contribute to a better understanding of the disease and how it affects patients' quality of life. This case-control study aims to retrospectively investigate the effect of AR on the quality of life of adult patients in Alahsa, Saudi Arabia.

## Materials and methods

This retrospective case-control study was conducted in the Alahsa region of Saudi Arabia between December 2024 and May 2025. The primary objective was to evaluate the impact of AR on quality of life, psychological distress, and insomnia severity among adults. Participants were recruited from Al Jabr Eye and ENT Hospital for the case group and from the general community in Alahsa for the control group.

The study population included adult Saudi residents aged 18 years and older. Participants were divided into two groups. The case group consisted of patients diagnosed with AR, identified through electronic medical records from Al Jabr Eye and ENT Hospital using the International Classification of Diseases, 10th revision (ICD-10) codes J30 (vasomotor and AR) and J30.4 (AR, unspecified). Inclusion criteria for cases included Saudi nationality, age ≥18 years, confirmed diagnosis of AR between January and December 2024 in the hospital system, and absence of comorbid chronic or psychiatric illnesses. Patients with conditions such as asthma, diabetes, hypertension, depression, or incomplete questionnaire data were excluded. The control group included individuals without a diagnosis of AR or any chronic illness. These participants were recruited from the general community using convenience sampling. Eligibility criteria for controls were Saudi nationality, age ≥18 years, absence of AR or chronic illness, and full participation in the study. Exclusion criteria included any physical or mental health condition or incomplete responses.

A total of 200 participants were enrolled in the study, including 50 cases and 150 controls. This sample size was calculated to detect the effect of AR on quality of life with 80% statistical power at a 95% confidence level (CI) and a 1:3 allocation ratio, using PASS software. From a list of 1,886 AR patients identified from hospital records using ICD-10 codes, 208 were selected via simple random sampling using STATA software for eligibility screening. After applying the inclusion and exclusion criteria, 50 participants were retained as cases. Controls were selected using convenience sampling from the general community and screened to match general demographic characteristics.

Participants completed three validated Arabic-language questionnaires distributed via Google Forms. The first was the General Health Questionnaire-12 (GHQ-12), developed by Goldberg in 1978, which includes 12 questions evaluating somatic symptoms, anxiety, social dysfunction, and depression. Each item is scored from 1 to 4, with higher scores reflecting poorer psychological health [14]. The second instrument was the AIS, which assesses sleep quality over the previous month through eight questions scored from 0 (no problem) to 3 (very serious problem) [15]. The third was the miniRQLQ, which includes 14 questions assessing seven symptom domains on a 7-point Likert scale, ranging from 0 (no disturbance) to 6 (very severe disturbance) [16]. Responses that were incomplete or duplicated were excluded from the final analysis.

This study involved human participants. Ethical approval was obtained from the Institutional Review Board (IRB) of the Alahsa Health Cluster under Log No. 33A-EP-2024, approved on 24 October 2024. Informed consent was obtained electronically from all participants before data collection.

Data analysis was performed using IBM SPSS Statistics (IBM Corp., Armonk, NY) for Windows, version 28. Descriptive statistics were used to summarize participant characteristics, with frequencies and percentages for categorical variables, and means and standard deviations (SD) for continuous variables. The chi-square test (χ²) was applied to compare categorical data between groups, and the exact probability test was used when expected frequencies were low. Independent sample t-tests were used to compare the mean GHQ-12 and AIS scores between cases and controls. Pearson correlation coefficients were calculated to assess the associations between miniRQLQ, GHQ-12, and AIS scores within each group. A p-value <0.05 was considered statistically significant.

## Results

Table 1 presents the sociodemographic characteristics of participants with AR (cases, n=50) and controls (n=150). The distribution of age, gender, occupation, housing, marital status, and smoking status did not show statistically significant differences between groups (p>0.05). Most participants across both groups were between 31 and 40 years old [cases: 17 (28.3%); controls: 43 (28.7%)]. The gender distribution was fairly balanced, with males accounting for 23 (46.0%) of the cases and 80 (53.3%) of the controls, while females represented 27 (54.0%) of the cases and 70 (46.7%) of the controls. Educational level was significantly associated with AR status (p=0.023), with a higher proportion of cases holding a bachelor’s degree or higher (33, 66.0%) compared to controls (64, 42.7%). Although not statistically significant, a higher number of AR cases were not working (22, 44.0%) and lived in villas or houses (37, 74.0%) compared to controls. Smoking status showed no significant difference, with non-smokers comprising the majority of both groups [cases: 44 (88.0%); controls: 125 (83.3%)].

**Table 1:** Sociodemographic characteristics of study cases with allergic rhinitis (cases) and controls P: Pearson X^2^ test; ^^^exact probability test; ^*^p<0.05 (statistically significant) SD: standard deviation

Demographics	Group				P-value
	Cases (n=50)		Controls (n=150)		
	N	%	N	%	
Age group, years					0.654
18-24	9	20.0%	36	80.0%	
25-30	9	25.7%	26	74.3%	
31-40	17	28.3%	43	71.7%	
41-50	11	30.6%	25	69.4%	
>50	4	16.7%	20	83.3%	
Mean ± SD	35.5 ± 11.2		34.6 ± 11.6		
Gender					0.369
Male	23	22.3%	80	77.7%	
Female	27	27.8%	70	72.2%	
Educational level					0.023^*^
Below secondary	4	21.1%	15	78.9%	
Secondary school	11	18.6%	48	81.4%	
Diploma	2	8.0%	23	92.0%	
Bachelor's degree/above	33	34.0%	64	66.0%	
Occupation					0.111^^^
Not working	22	34.4%	42	65.6%	
Student	9	26.5%	25	73.5%	
Employee	19	19.4%	79	80.6%	
Retired	0	0.0%	4	100.0%	
Housing					0.062^^^
Apartment	13	16.5%	66	83.5%	
Farm	0	0.0%	1	100.0%	
Villa/house	37	30.8%	83	69.2%	
Marital status					0.475^^^
Single	19	29.2%	46	70.8%	
Married	31	23.3%	102	76.7%	
Separated	0	0.0%	2	100.0%	
Smoking					0.640
Non-smoker	44	26.0%	125	74.0%	
Ex-smoker	1	12.5%	7	87.5%	
Current smoker	5	21.7%	18	78.3%	

Table 2 shows the distribution of responses to the GHQ-12 items, indicating that individuals with AR reported significantly higher psychological distress compared to controls (p<0.05 for all items). For example, only 14 (28.0%) of cases reported being able to concentrate "better than usual," compared to 123 (82.0%) of controls. Similarly, 18 (36.0%) of cases reported losing sleep "worse than usual," versus only 19 (12.7%) of controls. Emotional well-being also differed markedly; 23 (46.0%) of cases felt unhappy and depressed, compared to 118 (78.7%) of controls. Furthermore, negative self-perceptions were more prevalent among cases: only 33 (66.0%) viewed themselves as "better than usual" in terms of self-worth compared to 142 (94.7%) of controls. These consistent differences across all 12 items underscore a significantly greater psychological impact among AR patients.

**Table 2:** Distribution of responses to GHQ-12 items among allergic rhinitis cases and controls ^*^P<0.05 (statistically significant) GHQ-12: General Health Questionnaire-12

GHQ-12		Group				P-value
		Cases (n=50)		Controls (n=150)		
		No	%	No	%	
Been able to concentrate on what you’re doing	Better than usual	14	28.0%	123	82.0%	0.001^*^
	Same as usual	23	46.0%	24	16.0%	
	Worse than usual	11	22.0%	3	2.0%	
	Much worse than usual	2	4.0%	0	0.0%	
Lost much sleep over worry	Better than usual	14	28.0%	92	61.3%	0.001^*^
	Same as usual	14	28.0%	28	18.7%	
	Worse than usual	18	36.0%	19	12.7%	
	Much worse than usual	4	8.0%	11	7.3%	
Felt you were playing a useful part in things	Better than usual	18	36.0%	133	88.7%	0.001^*^
	Same as usual	22	44.0%	16	10.7%	
	Worse than usual	9	18.0%	1	0.7%	
	Much worse than usual	1	2.0%	0	0.0%	
Felt capable of making decisions about things	Better than usual	26	52.0%	132	88.0%	0.001^*^
	Same as usual	13	26.0%	15	10.0%	
	Worse than usual	11	22.0%	3	2.0%	
	Much worse than usual	0	0.0%	0	0.0%	
Felt constantly under strain	Better than usual	20	40.0%	93	62.0%	0.001^*^
	Same as usual	17	34.0%	33	22.0%	
	Worse than usual	8	16.0%	14	9.3%	
	Much worse than usual	5	10.0%	10	6.7%	
Felt you couldn’t overcome your difficulties	Better than usual	23	46.0%	126	84.0%	0.001^*^
	Same as usual	18	36.0%	17	11.3%	
	Worse than usual	8	16.0%	7	4.7%	
	Much worse than usual	1	2.0%	0	0.0%	
Been able to enjoy your normal day-to-day activities	Better than usual	25	50.0%	134	89.3%	0.001^*^
	Same as usual	14	28.0%	12	8.0%	
	Worse than usual	10	20.0%	3	2.0%	
	Much worse than usual	1	2.0%	1	0.7%	
Been able to face up to your problems	Better than usual	26	52.0%	125	83.3%	0.001^*^
	Same as usual	17	34.0%	19	12.7%	
	Worse than usual	7	14.0%	4	2.7%	
	Much worse than usual	0	0.0%	2	1.3%	
Been feeling unhappy and depressed	Better than usual	23	46.0%	118	78.7%	0.001^*^
	Same as usual	15	30.0%	21	14.0%	
	Worse than usual	11	22.0%	11	7.3%	
	Much worse than usual	1	2.0%	0	0.0%	
Been losing confidence in yourself	Better than usual	30	60.0%	132	88.0%	0.001^*^
	Same as usual	14	28.0%	13	8.7%	
	Worse than usual	5	10.0%	3	2.0%	
	Much worse than usual	1	2.0%	2	1.3%	
Been thinking of yourself as a worthless person	Better than usual	33	66.0%	142	94.7%	0.001^*^
	Same as usual	10	20.0%	4	2.7%	
	Worse than usual	7	14.0%	2	1.3%	
	Much worse than usual	0	0.0%	2	1.3%	
Been feeling reasonably happy, all things considered	Better than usual	30	60.0%	139	92.7%	0.001^*^
	Same as usual	15	30.0%	7	4.7%	
	Worse than usual	5	10.0%	3	2.0%	
	Much worse than usual	0	0.0%	1	0.7%	

The AIS responses revealed significantly higher sleep-related difficulties among AR cases compared to controls across all eight items (p<0.001 for each) (Table 3). Only 12 (24.0%) of cases reported no problem with sleep induction, compared to 85 (56.7%) of controls. Similarly, frequent awakenings during the night were reported as a considerable or very serious problem by 24 (48.0%) of cases, whereas only 12 (8.0%) of controls experienced the same. A markedly higher proportion of cases also reported issues with total sleep duration [35 (70.0%) vs. 27 (18.0%)] and overall sleep quality [34 (68.0%) vs. 23 (15.3%)]. Daytime consequences of poor sleep were also more prevalent among cases; for example, 32 (64.0%) of cases reported considerable or very serious problems with physical and mental functioning during the day, compared to only eight (5.3%) of controls.

**Table 3:** Distribution of AIS responses among allergic rhinitis cases and controls P: exact probability test; *p<0.05 (statistically significant) AIS: Athens Insomnia Scale

AIS		Group				P-value
		Cases (n=50)		Controls (n=150)		
		No	%	No	%	
Sleep induction (the time it takes you to fall asleep after turning off the lights)	No problem	12	24.0%	85	56.7%	0.001^*^
	Slight problem	12	24.0%	36	24.0%	
	Considerable problem	24	48.0%	25	16.7%	
	Very serious problem	2	4.0%	4	2.7%	
Awakenings during the night	No problem	15	30.0%	128	85.3%	0.001^*^
	Slight problem	11	22.0%	10	6.7%	
	Considerable problem	20	40.0%	6	4.0%	
	Very serious problem	4	8.0%	6	4.0%	
Final awakening earlier than desired	No problem	10	20.0%	110	73.3%	0.001^*^
	Slight problem	10	20.0%	20	13.3%	
	Considerable problem	25	50.0%	13	8.7%	
	Very serious problem	5	10.0%	7	4.7%	
Total sleep duration	No problem	14	28.0%	102	68.0%	0.001^*^
	Slight problem	1	2.0%	21	14.0%	
	Considerable problem	21	42.0%	18	12.0%	
	Very serious problem	14	28.0%	9	6.0%	
Overall quality of sleep (no matter how long you slept)	No problem	13	26.0%	113	75.3%	0.001^*^
	Slight problem	3	6.0%	14	9.3%	
	Considerable problem	17	34.0%	17	11.3%	
	Very serious problem	17	34.0%	6	4.0%	
Sense of well-being during the day	No problem	14	28.0%	125	83.3%	0.001^*^
	Slight problem	4	8.0%	16	10.7%	
	Considerable problem	21	42.0%	7	4.7%	
	Very serious problem	11	22.0%	2	1.3%	
Functioning (physical and mental) during the day	No problem	15	30.0%	130	86.7%	0.001^*^
	Slight problem	6	12.0%	12	8.0%	
	Considerable problem	17	34.0%	6	4.0%	
	Very serious problem	12	24.0%	2	1.3%	
Sleepiness during the day	No problem	14	28.0%	96	64.0%	0.001^*^
	Slight problem	4	8.0%	38	25.3%	
	Considerable problem	19	38.0%	10	6.7%	
	Very serious problem	13	26.0%	6	4.0%	

The miniRQLQ results showed that AR cases experienced significantly greater impairment in quality of life compared to controls across all measured domains (p<0.001 for all items). The most affected areas among cases were tiredness/fatigue (mean: 2.98, SD: 1.73), sleep difficulties (mean: 2.80, SD: 1.98), stuffy/blocked nose (mean: 2.62, SD: 2.15), and irritability (mean: 2.28, SD: 1.73). In contrast, controls reported minimal impact in these areas. Nasal and ocular symptoms such as sneezing, runny nose, itchy eyes, and sore eyes were also markedly more severe in cases. Additionally, daily activities, including work, recreation, and sleep, were substantially disrupted in the case group. These findings reflect the broad and significant impact of AR on patients’ physical comfort, emotional well-being, and day-to-day functioning (Table 4).

**Table 4:** Mean scores for miniRQLQ items among allergic rhinitis cases and controls P: independent sample t-test; *p<0.05 (statistically significant) miniRQLQ: mini Rhinoconjunctivitis Quality of Life Questionnaire; SD: standard deviation

miniRQLQ	Group				P-value
	Cases		Controls		
	Mean	SD	Mean	SD	
Regular activities at home and work	1.84	1.84	0.59	1.16	0.001^*^
Recreational activities	1.74	1.89	0.47	1.17	0.001^*^
Sleep (difficulties getting a good night's sleep and/or getting to sleep at night)	2.80	1.98	0.73	1.48	0.001^*^
Need to rub nose/eyes	1.84	2.16	0.25	0.84	0.001^*^
Need to blow the nose repeatedly	1.42	1.98	0.15	0.85	0.001^*^
Sneezing	1.58	1.80	0.17	0.79	0.001^*^
Stuffy blocked nose	2.62	2.15	0.30	0.99	0.001^*^
Runny nose	1.74	2.23	0.13	0.73	0.001^*^
Itchy eyes	1.04	1.94	0.17	0.64	0.001^*^
Sore eyes	0.78	1.63	0.09	0.52	0.001^*^
Watery eyes	0.52	1.23	0.27	0.97	0.001^*^
Tiredness and/or fatigue	2.98	1.73	1.19	1.69	0.001^*^
Thirst	1.70	2.29	0.51	1.12	0.001^*^
Feeling irritable	2.28	1.73	0.69	1.35	0.001^*^

**Figure 1:**
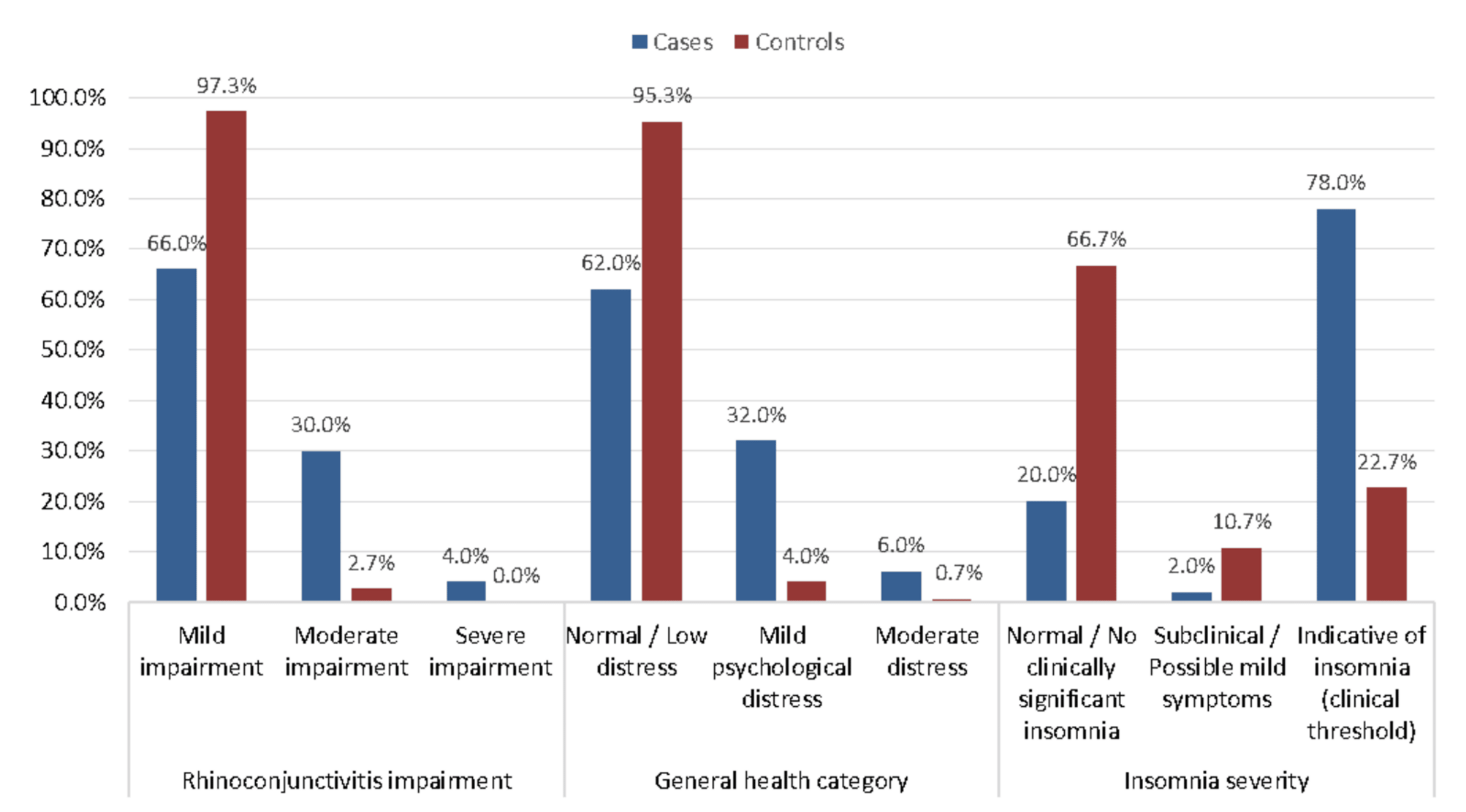
Distribution of quality-of-life impairment, psychological distress, and insomnia severity among allergic rhinitis cases and controls

Figure 1 illustrates that the distribution of quality-of-life impairment, psychological distress, and insomnia severity significantly differed between AR cases and controls (p<0.05 for all comparisons). A higher proportion of controls reported only mild rhinoconjunctivitis impairment (146, 97.3%) compared to cases (33, 66.0%), while moderate and severe impairment were notably more prevalent among cases [15 (30.0%) and two (4.0%), respectively]. Similarly, psychological distress was significantly greater in the case group, with only 31 (62.0%) categorized as having normal or low distress versus 143 (95.3%) of controls; 16 (32.0%) of cases experienced mild distress, and three (6.0%) had moderate distress. In terms of sleep health, 39 (78.0%) of cases met the clinical threshold for insomnia, compared to just 34 (22.7%) of controls, highlighting a substantial burden of sleep disturbance in individuals with AR.

**Figure 2:**
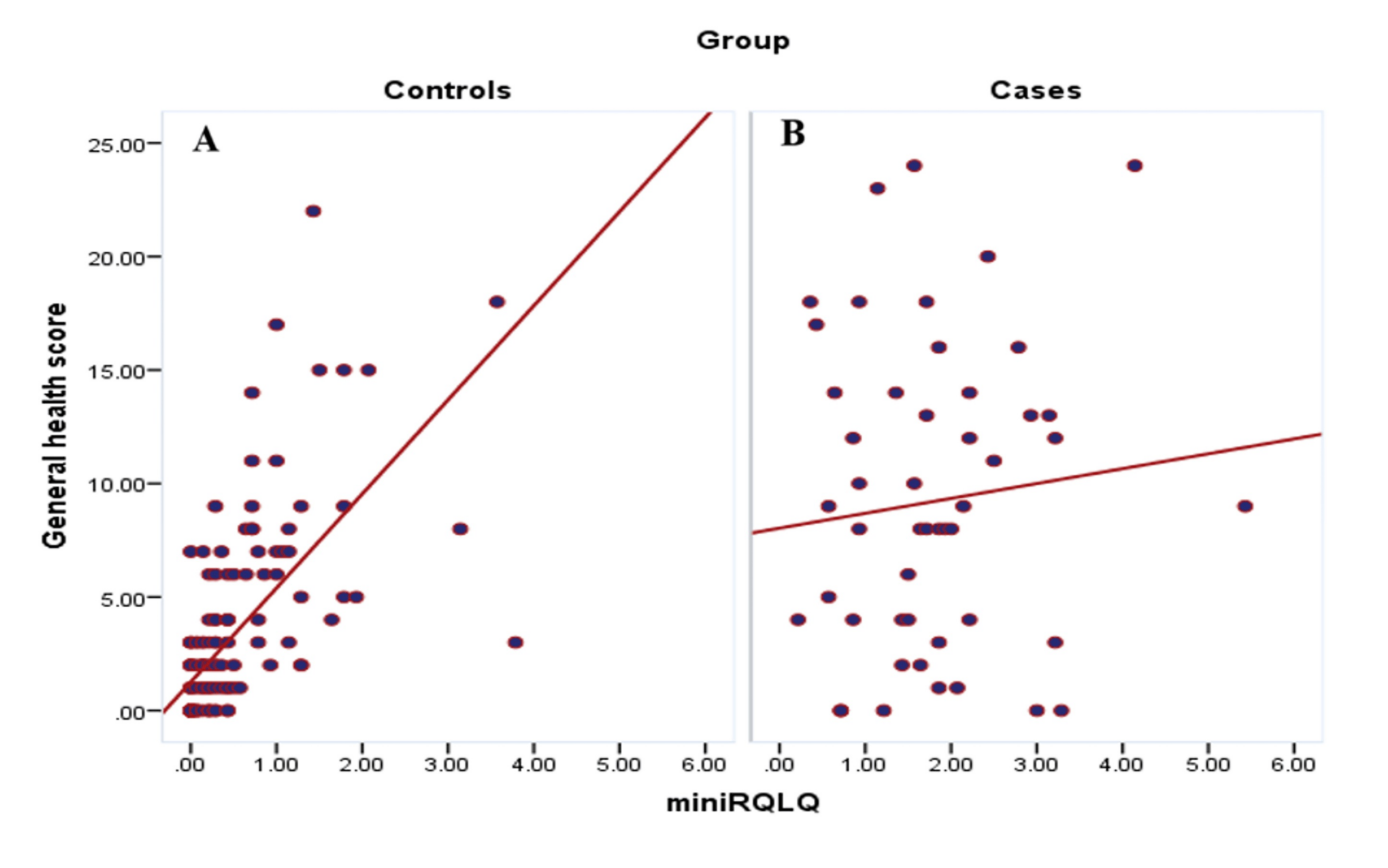
Correlation between rhinoconjunctivitis quality of life questionnaire and the general health score among study cases (A) and controls (B) r=0.1, p=0.493 for cases; r=0.7, p=.001 for controls miniRQLQ: mini Rhinoconjunctivitis Quality of Life Questionnaire

Figure 2 (A and B) illustrates the correlation between rhinoconjunctivitis quality of life scores and general health scores among study participants. Among cases, the correlation was weak and not statistically significant (r=0.1, p=0.493), indicating no meaningful relationship between allergic symptom burden and psychological distress. In contrast, a strong and statistically significant positive correlation was observed among controls (r=0.7, p=0.001), suggesting that in individuals without AR, poorer perceived quality of life due to rhinoconjunctivitis-like symptoms is closely associated with increased psychological distress.

**Figure 3:**
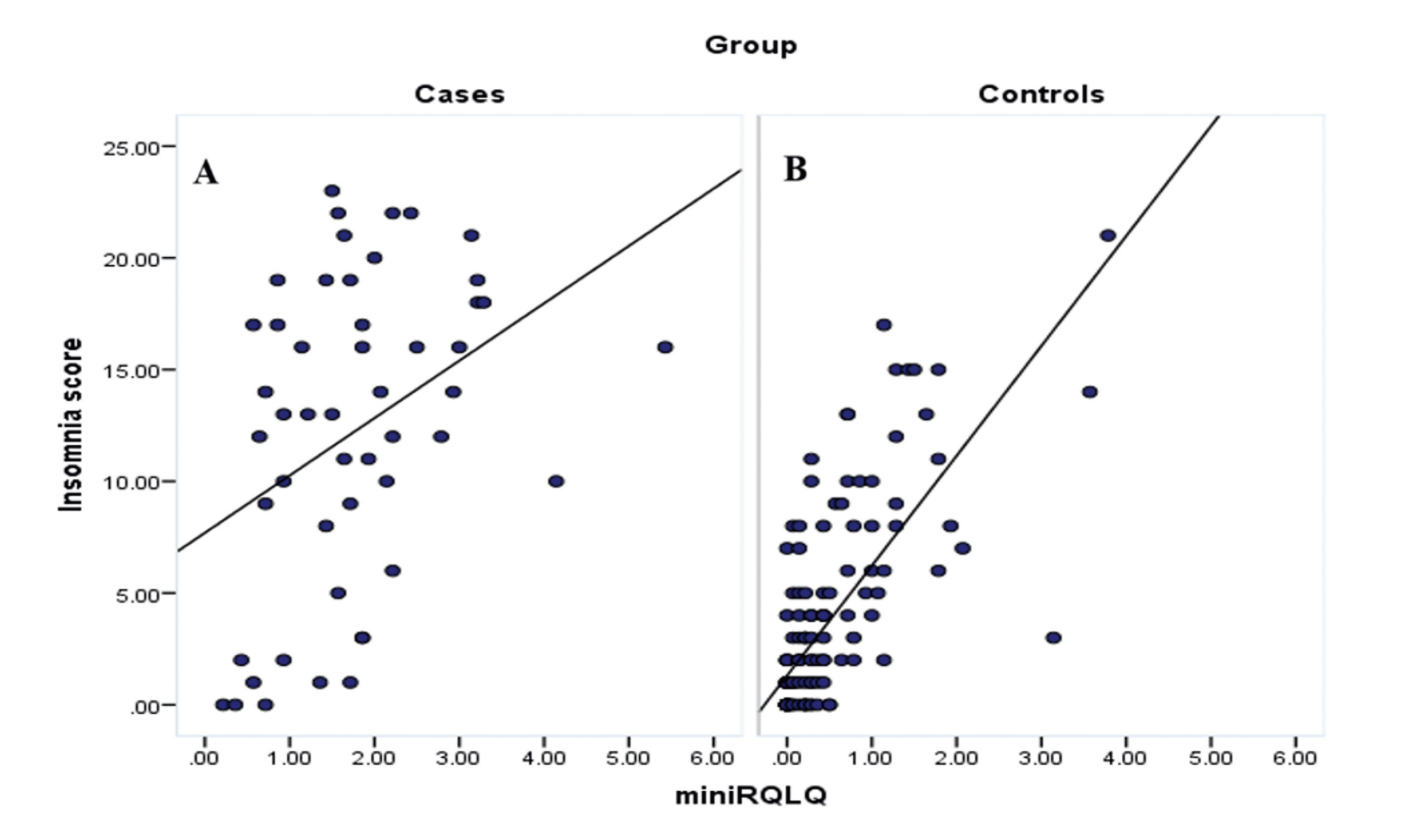
Correlation between rhinoconjunctivitis quality of life questionnaire and the AIS among study cases (A) and controls (B) r=0.4, p=0.006 for cases; r=0.7, p=0.001 for controls AIS: Athens Insomnia Scale; miniRQLQ: mini Rhinoconjunctivitis Quality of Life Questionnaire

Figure 3 (A and B) shows the correlation between rhinoconjunctivitis quality of life scores and insomnia severity, as measured by the AIS, among study cases and controls. Among cases, a moderate and statistically significant positive correlation was observed (r=0.4, p=0.006), suggesting that higher levels of rhinoconjunctivitis impairment are associated with greater severity of insomnia. For controls, a stronger and statistically significant correlation was found (r=0.7, p=0.001), indicating a robust relationship between quality-of-life impairment due to rhinoconjunctivitis and insomnia severity.

## Discussion

Our study showed that sociodemographic factors such as age, gender, occupation, housing type, marital status, and smoking habits do not significantly differ between individuals with and without AR in Alahsa, Saudi Arabia. This indicates that these variables may not play a major role in predisposing individuals to AR in this population, aligning with some previous studies that found no strong association between these factors and AR prevalence [17,18]. However, the higher proportion of AR cases among those with a bachelor’s degree or higher education suggests a potential link between higher educational attainment and AR. This observation may reflect increased awareness and diagnosis among educated individuals, as they might be more likely to seek medical attention for symptoms, or it could indicate environmental or lifestyle factors associated with higher education, such as increased exposure to indoor allergens or occupational settings [19,20].

The lack of significant differences in smoking status between cases and controls contrasts with some studies that have identified smoking as a risk factor for AR exacerbation, though not necessarily its prevalence [21]. The predominance of non-smokers in both groups may reflect cultural or regional trends in smoking behavior, which could explain the absence of an observed association. Additionally, the tendency of AR cases to reside in villas or houses, though not statistically significant, might suggest environmental influences such as greater exposure to household allergens like dust mites or pet dander, which are more common in larger living spaces [22]. The higher proportion of non-working individuals among AR cases could imply that symptom severity impacts employment status, or it may reflect differences in occupational exposures that were not fully assessed in this study [23].

Considering the relation with quality of life, the findings of this study showed that individuals with AR experience significantly higher psychological distress compared to healthy controls, as measured by the GHQ-12. This matches with both local and international studies that have documented the negative impact of AR on mental health. A study conducted in Tabuk found that AR patients had higher rates of anxiety and depression compared to the general population, supporting the notion that chronic nasal symptoms contribute to emotional distress [24]. Another study by Alhazmi et al. showed a significant reduction in the quality of life among patients with chronic rhinosinusitis (CRS), where the mean score for the Sino-Nasal Outcome Test-22 (SNOT-22) was 44.04 (SD: 25.38) compared with 26.82 (SD: 26.35) in nonpatients. Similarly, research in other Gulf countries, such as the UAE, has reported that AR is associated with poor sleep quality and impaired daytime functioning, which may exacerbate psychological symptoms [25].

Internationally, many studies have established a strong association between AR and reduced quality of life, including increased rates of depression, anxiety, and poor concentration. A large-scale European study found that AR patients were more likely to report feelings of worthlessness and sleep disturbances, mirroring the current findings [26]. Additionally, research in the United States has shown that AR-related sleep disruption contributes to daytime fatigue and cognitive impairment, which may explain the lower concentration levels observed in this study’s AR group [27]. The fact that only 28.0% of AR patients reported being able to concentrate "better than usual" (compared to 82.0% of controls) is particularly concerning, as it suggests that AR may impair work and academic performance, a finding consistent with studies from Asia and Europe and internationally [28,29,30].

The higher prevalence of emotional distress among AR patients in this study (46.0% feeling unhappy and depressed compared to 78.7% of controls) is also in line with global trends. A meta-analysis by Meltzer et al. (2009) found that AR patients were nearly twice as likely to experience depressive symptoms as non-AR individuals, reinforcing the bidirectional relationship between chronic allergic conditions and mental health [31]. Furthermore, the lower self-worth reported by AR cases (66.0% vs. 94.7% in controls) suggests that persistent symptoms may negatively affect self-perception, a phenomenon observed in studies from Australia and Japan, where AR patients reported lower confidence and social withdrawal due to their condition [32,33]. This is further supported by the distribution of disease burden across quality of life, psychological distress, and insomnia severity in our population. Compared to controls, a greater proportion of patients with AR reported moderate to severe levels of impairment in daily functioning and emotional well-being. A substantial number of cases experienced mild to moderate psychological distress, while the majority of controls fell within the normal range. Likewise, clinical insomnia was markedly more prevalent among AR cases, consistent with findings from previous research in Saudi Arabia.

The correlation analysis between quality-of-life scores and psychological distress revealed a weak and non-significant relationship among AR cases, suggesting that perceived symptom severity does not always correspond with psychological distress in chronic disease settings. In contrast, controls showed a much stronger and significant correlation, indicating that even low-grade symptoms may substantially affect psychological state in the general population. A similar pattern was observed between quality of life and insomnia severity, where both groups exhibited a significant positive correlation, reinforcing the well-documented link between allergic symptoms and sleep disruption. These results emphasize the importance of a comprehensive, multidisciplinary approach to AR management that includes mental health and sleep assessment.

While this study’s results are consistent with broader literature, some regional variations exist. For example, studies from Western countries often highlight the role of seasonal allergies in psychological distress, whereas in Saudi Arabia, perennial allergens (such as dust mites) may contribute to year-round symptoms, potentially worsening mental health impacts [34]. Additionally, cultural attitudes toward chronic illness and mental health in the Middle East may influence symptom reporting, with some patients underreporting psychological distress due to stigma [35].

Also, this study strongly indicates that AR is linked to significant sleep problems, as AR patients scored higher on the AIS compared to the control group. This is consistent with international research showing that AR often disrupts sleep. A US study found that over 60% of AR patients had sleep issues due to nasal congestion, similar to the 70% of AR patients in this study who reported shorter sleep duration [27]. European research also highlights that nighttime AR symptoms lead to fragmented sleep, which aligns with 48% of AR patients in this study experiencing frequent awakenings [28]. Difficulty falling asleep was also common among AR patients in this study, a finding supported by a study in Riyadh, Saudi Arabia, which noted nasal issues as a cause of prolonged sleep onset [36]. Moreover, studies from Japan and South Korea have shown that AR-related sleep disturbances can lead to daytime sleepiness and cognitive impairment, consistent with the 64% of AR patients in this study reporting daytime dysfunction [37,38]. The negative impact of poor sleep on daily life, including fatigue and irritability, is also supported by a multinational study [39].

The miniRQLQ assessment in this study clearly shows that AR significantly lowers quality of life across various aspects, notably causing fatigue, sleep problems, nasal blockage, and emotional irritability. These findings echo previous research both in Saudi Arabia and internationally, underscoring the wide-ranging impact of AR. For instance, a study in Riyadh found that around 60% of AR patients had moderate to severe sleep issues, which matches the significant sleep difficulties (mean: 2.80) reported in this study [40]. Many other studies have also assessed the effect of AR on daily activities and fatigue among cases [41,42].

This study has several limitations that should be acknowledged. Firstly, although cases were recruited from Al Jabr Eye and ENT Hospital, the main referral center for ENT care in the Alahsa region, relying on a single site may still limit the generalization of findings to the broader AR population. Additionally, the use of convenience sampling for controls could introduce selection bias and potential demographic mismatches. Second, due to its retrospective case-control design, the study cannot establish causal relationships between AR and observed impairments in quality of life, psychological well-being, or sleep; longitudinal studies are needed to better track symptom progression over time. Also, reliance on self-reported questionnaires introduces the possibility of recall and response bias, potentially affecting the accuracy of reported symptoms. Lastly, because the sample consisted solely of Saudi adults from a single geographic region, the generalizability of the findings to other populations or climatic settings may be limited.

## Conclusions

This case-control study demonstrated that AR significantly affects multiple aspects of quality of life among adults in Alahsa, Saudi Arabia. Patients with AR reported greater psychological distress, including symptoms of depression, anxiety, and impaired concentration, as measured by the GHQ-12. They also experienced more severe sleep disturbances, such as difficulty falling asleep and frequent awakenings, based on AIS scores. Furthermore, AR was associated with notable declines in physical comfort, emotional well-being, and daily activities, as captured by the miniRQLQ. These findings are consistent with previous literature and reinforce the classification of AR as a chronic condition with substantial health and socioeconomic impacts. The high burden of sleep and mental health symptoms among AR patients suggests that current management practices may not adequately address the full scope of AR-related challenges, pointing to the need for more integrated and patient-centered approaches.
